# Comparison of Three Regional Medicines Regulatory Harmonisation Initiatives in Africa: Opportunities for Improvement and Alignment

**DOI:** 10.34172/ijhpm.2024.8070

**Published:** 2024-05-05

**Authors:** Tariro Sithole, Nancy Ngum, Mercy Owusu-Asante, Stuart Walker, Sam Salek

**Affiliations:** ^1^School of Life and Medical Sciences, University of Hertfordshire, Hatfield, UK.; ^2^African Union Development Agency-New Partnership for Africa’s Development (AUDA-NEPAD), Johannesburg, South Africa.; ^3^Food and Drug Authority Ghana, Accra, Ghana.; ^4^Centre for Innovation in Regulatory Science, London, UK.; ^5^Institute for Medicines Development, Hatfield, UK.

**Keywords:** Medicines Regulatory Harmonisation, African Medicines Agency, EAC, ZaZiBoNa, SADC, ECOWAS

## Abstract

**Background:** The African Medicines Regulatory Harmonisation (AMRH) Initiative was formed in 2009 and subsequently, three regional initiatives (East African Community Medicines Regulatory Harmonisation [MRH], Southern African Development Community [SADC]/ZaZiBoNa MRH, and the Economic Community of West Africa States MRH) were established. As these initiatives serve as a foundation for the African Medicines Agency (AMA), the aim of this study was to compare their operating models, successes and challenges to identify opportunities for improvement and alignment.

**Methods:** A mixed method approach was used for the data collection using a questionnaire, the Process, Effectiveness and Efficiency Rating (PEER), developed by the authors specifically for this study and semi-structured interview techniques. There were 23 study participants (one from each agency of the member countries of the three regions). It was hoped that data generated from this study would lead to a series of recommendations, which would then be ratified by the regulatory authorities.

**Results:** Most respondents stated that AMRH contributed to the strengthening of regulatory systems and harmonising regulatory requirements across economic regions of Africa, potentially resulting in improved access to quality-assured medicines. Although established at different times and at the discretion of each region, the marketing authorisation application review processes are largely similar, with a few differences noted in the eligibility and submission requirements, type of procedures employed, the timelines and fees payable. The challenges identified in the three regions are also similar, with the most noteworthy being the lack of a binding legal framework for regional approvals.

**Conclusion:** In this study, we compared the process, successes and challenges of these three regional harmonisation initiatives in Africa addressing the areas of legal frameworks, information management systems, the accessibility and affordability of medicines and reliance that will bring greater alignment and efficiency in their operating models, thereby strengthening the foundation of the soon-to-be-operationalised AMA.

## Background

**Key Messages**

**Implications for policy makers**

- Information is needed regarding the operating models and successes and challenges experienced to date for the three initiatives for medicines regulation established in the economic communities of Africa under the auspices of the African Medicines Regulatory Harmonisation (AMRH) Initiative.
- Qualitative questionnaire and literature search data reveal that the marketing authorisation application review processes of the three Medicines Regulatory Harmonisation (MRH) programmes, the East African Community; Southern African Development Community (SADC)/ZaZiBoNa; and Economic Community of West African States are largely similar, with a few differences noted in the eligibility and submission requirements, type of procedures employed (eg, centralised or decentralised), the timelines and fees payable.
- Participants uniformly agreed that harmonisation of regulatory requirements, information sharing and capacity building are the primary benefits of the MRH initiatives, whilst the principal challenges of the programmes are a lack of centralised submission and tracking and inconsistency in stringency of submission requirements.
- Recommendations to mitigate these challenges include the alignment of operating models; development of a regional legally binding framework to allow establishment of a centralised procedure; formation of information management systems and support of capacity strengthening to facilitate mutual recognition and reliance.
- The recommendations made in this study will bring greater alignment and efficiency to the operating models of the three regional harmonisation initiatives, strengthening the foundation of the soon to be operationalised African Medicines Agency (AMA).

**Implications for the public**

Since 2009, the African Medicines Regulatory Harmonisation (AMRH) Initiative has made significant gains in strengthening national regulatory systems and harmonising regulatory requirements to bring needed, quality-assured medicines to the African people. However, as the COVID-19 public health emergency highlighted, achieving the expedited regulatory review of medicines and vaccines is vital to shorten the time to market various life-saving medical products. Work must therefore continue to achieve the objectives of shorter timelines and simultaneous access to various African markets, including the recommendations of this study chiefly, the development of legally binding frameworks for regulatory review and increased reliance and collaboration among African regulatory authorities.

It is the responsibility of national medicines regulatory authorities (NMRAs) to ensure that medical products such as medicines and vaccines used by the public are of good quality, safe and effective.^1^ The role of NMRAs was brought into the spotlight during the COVID-19 pandemic, as these agencies were responsible for the review and approval of novel vaccines in the shortest possible time. This public health emergency resulted in an increase in the use of reliance and collaborative registration pathways among regulatory authorities, as they sought to shorten the time to market various life-saving medical products.^2^

**Figure 1:**
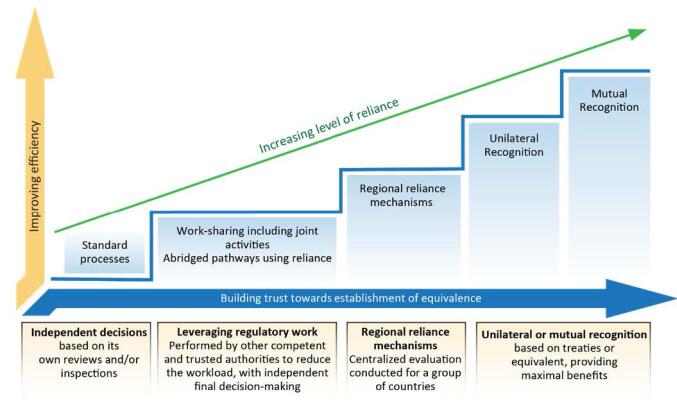
Key Concepts and Levels of Reliance (WHO, 2021).^4^

 Reliance is defined by the World Health Organization (WHO) as “the act whereby the regulatory authority in one jurisdiction takes into account and gives significant weight to assessments performed by another regulatory authority or trusted institution, in reaching its own decision” (Figure 1).^3,4^ The foundation for NMRA use of reliance was built prior to the COVID-19 pandemic, when NMRAs invested in implementing reliance principles to improve efficiency and establish the relevant systems in accordance with the WHO good reliance practices guidelines.^3,5^ A type of reliance is joint review or work sharing, in which the review or assessment of a medicine is conducted by two or more NMRAs collaboratively. Examples of joint review or work-sharing initiatives are the East African Community Medicines Regulatory Harmonisation (EAC MRH) initiative, the ZaZiBoNa/Southern African Development Community Medicines Regulatory Harmonisation (SADC MRH) initiative and the Economic Community of West African States Medicines Regulatory Harmonisation (ECOWAS MRH) initiative currently implemented in Africa through the African Medicines Regulatory Harmonisation (AMRH) Initiative established in 2009.^6^

 Whilst individual NMRAs in Africa have the opportunity to review products independently, there are currently five major regional initiatives that were designed to bring groups of NMRAs together, in order to expedite patients’ access to medicines and make recommendations for registration to the individual NMRAs. However, an NMRA can be involved in more than one regional initiative due to their geographical position. The three major regional initiatives in Africa are ZaZiBoNa, the EAC-MRH and the ECOWAS MRH, which have been evaluated and compared. In these regions, because there is not an established legal framework, the recommendations are not mandated as would be the situation for a centralised procedure. Neither is there mutual recognition, which would be the situation with a decentralised procedure, as is exemplified in the European Medicines Agency (EMA).

###  The East African Community Medicines Registration Harmonisation Initiative

 The EAC MRH initiative was established in 2012 as a 5-year pilot and the first regulatory harmonisation project under the AMRH, with the overarching goal to improve access to quality medicines and to test the feasibility of regulatory harmonisation in Africa.^7^ Participating countries were Burundi, Kenya, Rwanda, South Sudan, Tanzania, and Uganda.^8^ The beginning model employed by the EAC involved NMRA staff from participating countries travelling to Copenhagen to participate in joint assessment sessions with the WHO Prequalification of Medicines programme.^7^ However, this model was later discontinued due to unsustainability and assessment sessions are now held within the EAC region. In the current model employed by the EAC, lead NMRAs are designated for key functions: Tanzania for medicines evaluation and registration, Uganda for good manufacturing practices inspections, Rwanda for information management systems, and Kenya for quality management systems.^7^ Therefore, products are submitted to the Tanzania NMRA, which conducts the validation and primary review of the application before presenting it to the joint assessment session, which is attended by a representative from each country for further consideration. Only after a recommendation is issued, will the applicant be expected to submit individual applications for marketing authorisation and a fee to each NMRA.^8^ Marketing authorisations are granted individually by each country.

 The Tanzania NMRA was the first in Africa to attain maturity level 3 status in the WHO Global Benchmarking Tool (GBT) programme in 2018.^4^ Maturity level 3 indicates a stable and well-functioning regulatory system.^9^

###  ZaZiBoNa /Southern African Development Community Medicines Regulatory Harmonisation Initiative

 ZaZiBoNa was founded in 2013 by Zambia, Zimbabwe, Botswana, and Namibia to address the challenges of long registration times and inadequate capacity and resources in these countries.^10^ In 2015, the SADC MRH project was launched, absorbing ZaZiBoNa. Membership has since grown to include all 16 SADC countries (9 active members, 5 non-active members, and 2 observers). Active member status is determined by the capacity to conduct assessments and good manufacturing practice inspections and the active member countries are Botswana, Democratic Republic of Congo, Malawi, Mozambique, Namibia, South Africa, Tanzania, Zambia, and Zimbabwe.^10^ The SADC MRH initiative does not have centralised submissions or approvals/registrations due to the absence of a regional legal framework. In the current model, applicants simultaneously submit applications for registration and pay fees to each of the countries in which they wish to market their medicinal products.^6,10^ To be eligible for joint assessment, applications should be submitted to a minimum of two countries. The assessment of dossiers/applications is carried out using a rapporteur and co-rapporteur before consideration of the report by a group of assessors from all the active member countries. Once the evaluation is concluded, an assessment report with a recommendation and a consolidated list of questions is produced and communication of the list of questions to the applicants as well as the final decision on the registration/marketing authorisation of the medicinal products is left to the individual participating countries.^10^ Two SADC MRH NMRAs have attained WHO GBT maturity level 3 status, Tanzania, as previously mentioned, and South Africa in 2022.^9,11^

###  Economic Community of West African States Medicines Regulatory Harmonization Initiative

 Similar to other regions in Africa, the ECOWAS region faced challenges in technical capacity and financial resources. In addition, because the ECOWAS region comprises Portuguese-, English-, and French-speaking countries,^12^ the differences in official national language further complicated and delayed the implementation of harmonisation. The ECOWAS MRH initiative was launched in 2017 by the West African Health Organization (WAHO) to improve the availability of high-quality, safe and effective medicines and vaccines in ECOWAS.^13^ The ECOWAS MRH initiative aimed to reduce the time to registration and improve regulatory oversight through jointly registering locally manufactured and imported medical products.^12^ Although the ECOWAS MRH initiative was launched in 2017, joint assessments commenced in 2019 and to date, seven NMRAs; that is, Burkina Faso, Cote d’Ivoire, Ghana, Nigeria, Senegal, Sierra Leone, and Togo have participated in the sessions. Although these seven countries participate in the joint assessments, the outcomes are taken as a basis for the regulatory decision in all 15 NMRAs in the ECOWAS region.^13^ In the model employed by the ECOWAS MRH, a country is appointed to serve as lead NMRA/coordinator for two years on a rotational basis. This lead NMRA is assigned to serve as coordinating agency for product applications and is responsible for receiving, validating and preparing applications for review by an assessment team comprising staff from the seven participating NMRAs. The report is then considered during the joint assessment session of the expert working group. The WAHO Secretariat serves as an administrative agency responsible for issuing notifications of recommendations at the regional level. Once this process is completed, each NMRA that receives an application for a jointly reviewed product implements their national procedure to issue a national marketing authorisation. Applicants are given a maximum of two years after the regional review to submit applications for marketing authorisation to countries of their choice. Two ECOWAS NMRAs attained WHO GBT maturity level 3 status Ghana in 2020 and Nigeria in 2022.^11,14^

 A common challenge for all three regions implementing harmonisation initiatives was the varying regulatory capacities of participating countries. Barton and colleagues suggested three factors that may be more important: “(1) fragmented and complex drug regulations, (2) suboptimal enforcement of existing regulations, and (3) poorly designed disincentives for non-compliance.”^15^ To address this issue, capacity building was included in the regional activities to improve standards, build trust and facilitate the proposed harmonisation and reliance initiatives. The AMRH was posited as a precursor to the African Medicines Agency (AMA), which is in the process of being established as a specialised agency of the African Union to improve access to high-quality, safe and efficacious medical products in Africa.^5^ It is therefore timely and necessary to conduct a comparison of the existing regional harmonisation initiatives to identify opportunities for improvement and alignment.

###  Study Objectives

- Compare the operating model, review process and requirements of the three harmonisation initiatives;
- Compare the successes and challenges of the initiatives;
- Identify opportunities for improvement and alignment of the initiatives and develop recommendations for the way forward.

## Methods

###  Study Participants 

 All seven members of the EAC MRH (Burundi, Kenya, Rwanda, South Sudan, Tanzania, Uganda, and Zanzibar) as well as all nine active members of the ZaZiBoNa/SADC MRH (Botswana, Democratic Republic of Congo, Malawi, Mozambique, Namibia, South Africa, Tanzania, Zambia, and Zimbabwe) and all seven members of the ECOWAS MRH (Burkina Faso, Cote d’Ivoire, Ghana, Nigeria, Senegal, Sierra Leone, and Togo) participated in the three initiatives that were used for this comparative study. Each regulatory authority was asked to nominate one individual for completing the questionnaire, who had the responsibility for monitoring and documenting regulatory performance metrics.

###  Content Validity of the PEER Questionnaire 

 Data were collected in 2021 and 2022 using the Process, Effectiveness and Efficiency Rating questionnaire (PEER) developed by the authors. In order to further ascertain the content validity of the PEER questionnaire the respondents were asked to answer seven questions with a “yes or no” response options following completion of the PEER questionnaire (Supplementary file 1, Box 1): Did you find the questions clear and straightforward to respond?; Did you find the response options relevant to the heading of each section (A to E)?; Did you find the questions relevant to the aims and objectives of the study?; Did you find the questions relevant to your authority and work-sharing initiative?; Did you find any relevant questions missing? If yes, please state which questions were missing in the space provided after this list of questions; Did you find any questions that should be excluded? If yes, please state the questions that should be excluded in the space after this list of questions; Did you find the questionnaire useful to reflect on both your agency experience as well that of the initiative?

 In addition, as part of the cognitive debriefing aspect of the content validity and triangulation of the responses to the PEER questionnaire, semi-structured interviews were carried out with the original survey respondents, and this was designed specifically in order to fulfil the trustworthiness criteria such as credibility, confirmability, dependability and transferability by clarifying respondents’ answers and confirming that they had fully understood the questions and their answers.

 Furthermore, the rigour and quality of the qualitative part of our study was tested including: credibility, through close and maintained engagement with the respondents (ie, focal person) and triangulation; confirmability, through involving the head of each authority by checking the responses of the “focal person” and the research and keeping notes of the course of events; dependability, through keeping written accounts of the qualitative research process; and transferability, through detailed and comprehensive step-by-step description of the structure and procedure and their operationalisation.^16-18^

###  Data Collection

 The PEER questionnaire was completed by the focal person/assessor in each country and validated by the head of the authority. The questionnaire comprised five sections under the headings *Demographics*; *Benefits*; *Challenges*; *Improving the performance (effectiveness and efficiency) of the work-sharing programme;* and *Envisaging the strategy for moving forward*. Data were also extracted from the literature.

 Based on the synthesis of the results, it was hoped that the authors would generate a series of recommendations, which would then be presented to the regulatory agencies for their endorsement.

 The PEER questionnaire was developed and validated by the authors in association with the regulatory authorities specifically for this study. It was piloted with two regulatory authorities in each of three regions who were given the opportunity to comment on the content and the relevance of the questionnaire using a 7-item checklist (Supplementary file 1, Box 1). As part of the relevance aspect of their evaluation they were asked to comment on what was missing and what should be deleted (as not relevant) from the questionnaire. As a result, minor changes were implemented and the final version of the PEER questionnaire was constructed. The study participants were then given two weeks to complete the questionnaire, and two reminders were sent out subsequently so that the data from all participating regulatory authorities were completed within the month after initiation. It was suggested that the questionnaire, which was sent out to the participants by e-mail, could be completed in 15 minutes (average time taken to complete during the pilot) and returned by e-mail as an attachment. Furthermore, we used a triangulation approach in this study, employing multiple methods of data generation including online Zoom virtual interviews in order to ascertain the accuracy of the study participants’ responses as well as to develop a comprehensive understanding of the phenomena being explored.

###  Data Processing and Analysis

 The study was exploratory (hypothesis generating) and the nature of the data generated through the PEER questionnaire and the interviews (which were transcribed verbatim) was qualitative. The content analysis technique was used to analyse the qualitative (text) data. The content analysis of the qualitative data employed a conventional approach, using inductive coding based on the data, from which a set of cohesive themes were then generated.

 An initial meeting (TS, NN, MOA, SW, and SS) was conducted to examine the content of the data collected and identify initial concepts across the different forms of data collected. Data in the form of key phrases, statements, lists, were independently extracted from the PEER Questionnaire and transcribed texts. A thematic analysis was undertaken where three members of the core team (TS, NN, and MOA) familiarised themselves with the different forms of data and added initial codes.^19^ Constant comparison across the different forms of data informed an initial thematic framework to enable consistent coding of the data. If themes were identified from the data that did not fit the initial coding framework, a new code was established to involve the theme in the analysis.

 The researchers (TS, NN, and MOA) worked independently to identify themes, but met to discuss the themes and establish consensus. All themes, particularly where consensus could not be achieved, were further discussed and agreed with the rest of the research team (NN, MOA, and SW). This enabled analysis codes to be modified as new ideas were developed.^19^ All members of the core research team (TS, NN, MOA, SW, and SS) then commented on the proposed themes and supporting evidence. Reliability was therefore established through discussion, and findings were based on researcher agreement.^20,21^

 Descriptive statistics such as frequency were used to analyse the nominal data.

## Results

###  Study Participants Characteristics and Response Rate 

 Each regulatory authority nominated a focal person who was responsible for measuring and monitoring regulatory performance of their respective region. Each focal person from the seven members of the EAC MRH (Burundi, Kenya, Rwanda, South Sudan, Tanzania, Uganda, and Zanzibar) as well as all nine active members of the ZaZiBoNa/SADC MRH (Botswana, Democratic Republic of Congo, Malawi, Mozambique, Namibia, South Africa, Tanzania, Zambia, and Zimbabwe) and all seven members of the ECOWAS MRH (Burkina Faso, Cote d’Ivoire, Ghana, Nigeria, Senegal, Sierra Leone, and Togo) completed the PEER questionnaire and took part in the interview, resulting in a 100% (ie, 23 respondents) response from each of the regions.

###  Part I: Requirements and Review Process

 A comparison of the three harmonisation initiatives was conducted (Table).

**Table:** Comparison of the Review Process and Requirements for MRH of the EAC, ZaZiBoNa/SADC, and ECOWAS Initiatives

	**EAC-MRH**	**SADC MRH/ZaZiBoNa**	**ECOWAS MRH **
Type of procedure	Decentralised; however, there is no flexibility in selection of lead NMRA which is the equivalent of the Reference Member State and the EAC Secretariat serves as an administrative agency	Hybrid of decentralised and centralised; implementing NMRA serves as a coordinating agency	Hybrid of centralised and decentralised procedure; WAHO Secretariat serves as an administrative agency and the lead NMRA serves as coordinating agency
Legally binding framework	None	None	None
Eligibility criteria for joint review	Previous intention to market in all participating countries, currently minimum of 2 countries	Submission to a minimum of 2 countries	None, as the regional review precedes national submissions; however, applicants are encouraged to market their products in all 15 countries
Submission windows	No windows; open throughout the year	No windows; open throughout the year	Four 30-day submission windows (Feb, May, Jul, Oct)
Submission of applications	Submission to the lead NMRA then submission to the remaining countries of interest immediately once the regional joint review is completed	Submission to all countries applicant is interested in marketing the product before the regional joint review commences	Submission to lead NMRA based on published expression of interest after a pre-submission meeting, then submission to the remaining countries of interest within 2 years of the regional joint review being completed
Assessment/review process	Primary and peer review by lead NMRA, peer and final review at joint assessment session. Primary review by rapporteur selected using applicable criteria, peer review by second country (co-rapporteur), final review at joint assessment session	Primary review by assessment team, peer and final review by expert working group at joint assessment session	
Communication with sponsors	Responsibility of EAC Secretariat	Responsibility of each individual country to which the application was submitted	Responsibility of WAHO Secretariat
Final approval and marketing status	Approval issued by each individual NMRA in receipt of application and marketed only in those countries	Approval issued by each individual NMRA in receipt of application and marketed only in those countries	Approval issued by each individual NMRA in receipt of application and marketed only in those countries
Target timelines	315 days including applicant’s time from the date validation is completed to the date of regional recommendation	270 days including applicant’s time (from the date validation is completed to the date of regional recommendation)	226 days including applicant’s time (from the date validation is completed to the date of regional recommendation)
Target timeline for registration by NMRA after a regional recommendation	90 days	90 days	90 days
Fees	Paid to each individual NMRA; however, there are plans to pilot an additional regional fee	Paid to each individual NMRA; however, there are plans to pilot an additional regional fee	Regional fee paid to the WAHO Secretariat and the lead NMRA and a national fee paid to each NMRA where a national application is made

Abbreviations: EAC, East African Community; ECOWAS, Economic Community of West African States; MRH, Medicines Regulatory Harmonisation; NMRA, national medicines regulatory agencies; SADC, Southern African Development Community; WAHO, West African Health Organization.

####  Type of Procedure

 The EAC MRH employs a decentralised procedure in which the applicant does not have the flexibility to choose the country to act as lead NMRA or reference member state for their application. The lead NMRA for all applications submitted to the EAC MRH is the Tanzania NMRA. In comparison, the ZaZiBoNa/SADC MRH employs a hybrid of the decentralised and centralised procedures in that the submission and final approval of applications are decentralised, while the review or assessment is centralised with the implementing NMRA; that is, Zimbabwe, serving as a coordinating agency that assigns applications to a rapporteur and co-rapporteur. Similarly, the ECOWAS MRH employs a hybrid of the centralised and decentralised procedures in that the process begins with a centralised joint regional review coordinated by the lead NMRA (currently Nigeria and rotated on a 2-year basis) and supported administratively by the WAHO Secretariat. The process is then decentralised, with each NMRA implementing a national procedure to issue national marketing authorisation upon receipt of applications for the jointly reviewed products.

####  Legally Binding Framework

 The EAC MRH, ECOWAS MRH, and ZaZiBoNa/SADC MRH all do not have legally binding frameworks; therefore, approvals are issued at country level and the products can only be marketed in those specific countries.

####  Eligibility Criteria

 The ECOWAS MRH does not have eligibility criteria because the regional review precedes national submissions; however, applicants are encouraged to market their products in all 15 countries, whereas for the EAC MRH and ZaZiBoNa/SADC MRH, the eligibility criteria is submission (or intention to submit for EAC MRH) to a minimum of two countries to be considered for joint regional review.

####  Submission Windows

 The EAC MRH and ZaZiBoNa/SADC MRH are open for submission of applications all year round, while the ECOWAS MRH accepts applications in four windows each year; that is, February, May, July, and October for 30 days.

####  Submission of Applications

 For the EAC MRH and ECOWAS MRH, applications are submitted to the lead NMRA first then to the remaining countries of interest once the assessment is completed. For the ZaZiBoNa/SADC MRH, applications are submitted only to countries where the applicant is interested in marketing the product.

####  Assessment/Review Process

 The primary review and peer review of applications submitted to the EAC MRH is conducted by the lead NMRA before a final review by all seven EAC countries at a joint assessment session, while for the ZaZiBoNa/SADC MRH, the primary review and peer review is conducted by a rapporteur and co-rapporteur assigned for that particular application before a final review by all nine active member states at a joint assessment session. For the ECOWAS MRH, the primary review is conducted by an assessment team constituting the seven ECOWAS MRH countries before a peer and final review by the expert working group at a joint assessment session of the seven participating countries.

####  Communication With Sponsors

 The responsibility for communication with applicants lies with the EAC Secretariat for the EAC MRH and the WAHO Secretariat for the ECOWAS MRH. For the ZaZiBoNa/SADC MRH, communication with applicants is carried out by each individual country to which the application was submitted.

####  Final Approval and Marketing Status

 The final approval is issued by each individual NMRA in receipt of the application and marketed only in those countries in all three regions.

####  Target Timelines 

 The target timeline for the EAC MRH from the date validation is completed to the date of final regional recommendation is 315 days, inclusive of the applicant’s time. Applicants are then expected to immediately submit applications to the countries in which they wish to market their products and be issued with a marketing authorisation within 90 days from the date of the regional recommendation. The ECOWAS MRH has a similar process and the target timeline from the date validation is completed to the date of final regional recommendation is 226 days inclusive of the applicant’s time. Applicants are then given up to 2 years to submit applications to the countries in which they wish to market their products. The target time for the countries to issue a marketing authorisation once they receive an application is within 90 days. The target timeline for ZaZiBoNa/SADC MRH from the date an application is first discussed at an assessment session to the date a final regional recommendation is given is 270 days, inclusive of the applicant’s time. Since the applications are submitted to each individual country in which the applicant wishes to market their products before the joint review, countries are expected to issue the marketing authorisation within 90 days of the regional recommendation.

####  Fees

 Fees are paid to the individual NMRA for registration in each country of interest in all three initiatives. In the ECOWAS MRH, this is preceded by payment of a regional fee to the WAHO Secretariat for the regional review. There are plans to pilot a regional fee in both the EAC MRH and ZaZiBoNa/SADC MRH in the near future. The regional application fees are intended to be used to finance joint reviews in addition to other sources of income, such as partners’ support and self-funding by the participating countries in some of the regions.

###  Part II: Successes

 For the comparisons in this section, a vote by the majority of countries (>50%) in a region is recorded as a vote by the region.

**Figure 2:**
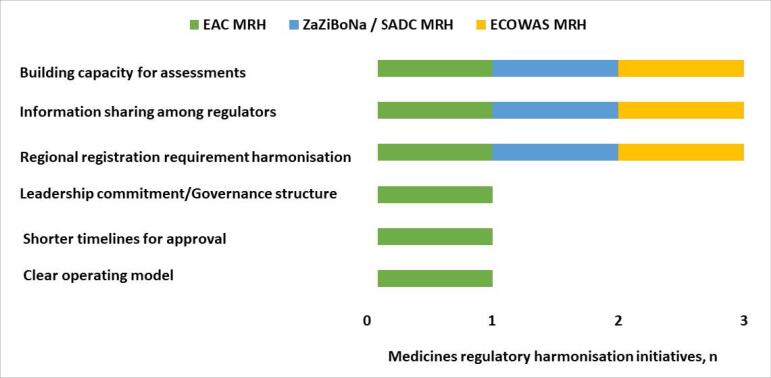
Strengths of the Medicines Regulatory Harmonisation Initiatives. Abbreviations: EAC, East African Community; ECOWAS, Economic Community of West African States; MRH, Medicines Regulatory Harmonisation; SADC, Southern African Development Community.

 There is agreement in the three MRH initiatives about the following strengths of the MRH program; harmonisation of registration requirements across the region, information sharing among regulators and the building of capacity for assessments. However, leadership commitment/governance structure, clear operating model and shorter timelines for approval were identified as strengths only by the EAC MRH (Figure 2).

**Figure 3:**
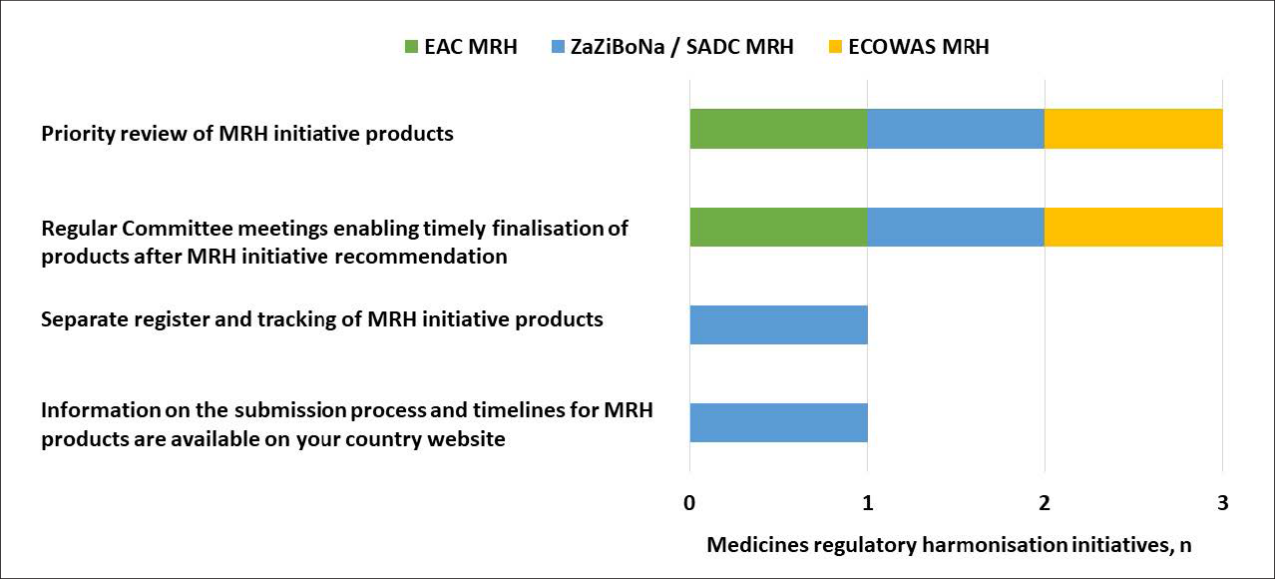
Strength of Country Processes in Implementing the Medicines Regulatory Harmonisation Programme. Abbreviations: EAC, East African Community; ECOWAS, Economic Community of West African States; MRH, Medicines Regulatory Harmonisation; SADC, Southern African Development Community.

 In all three initiatives, the review of MRH initiative products is prioritised and Committee meetings held regularly enable the timely finalisation of products after an MRH recommendation. These are the strengths of the country processes in the majority of countries. However, none of the MRH initiatives have a list of the products approved using joint reviews available on the individual country websites and only ZaZiBoNa/SADC MRH have information on the submission process and timelines for MRH products available on the majority of individual country websites as well as a separate register and tracking of MRH products (Figure 3).

####  Medicines Regulatory Harmonisation Benefits to Member Countries (Regulators)

 There is consensus from all three MRH initiatives on the benefits received by member countries (regulators) from participating in the MRH programme and these are the training, which has improved the performance of the assessors, enabling the application of high standards of assessment regardless of the size of the country or maturity of the regulatory authority. This platform has also made it easier for information and knowledge exchange among the countries. However, only EAC MRH were of the view that the shared workload resulted in shorter timelines for approval compared with the individual timelines of the majority of EAC countries.

####  Medicines Regulatory Harmonisation Benefits to Manufacturers (Applicants)

 There is agreement in all three regions about the benefits of the MRH programme for manufacturers/applicants and these are the reduction of the burden of preparing multiple dossiers, as under the MRH programme, only one dossier (modules 2 -5) is compiled for submission to multiple countries. Other benefits are the saving in time and resources, as applicants receive the same list of questions from multiple countries enabling compilation of a single response package as well as simultaneous access to various market. However, only the EAC MRH were of the view that applicants benefited from shorter timelines for approval under the MRH programme compared with the individual timelines of the majority of EAC countries.

####  Medicines Regulatory Harmonisation Benefits to Patients

 The consensus amongst the three regions was that the MRH programme has resulted in quicker access and increased availability of quality-assured medicines for patients; however, this was not at a reduced price.

###  Part III: Challenges

 For the comparisons in this section, a vote by the majority of countries (>50%) in a region is recorded as a vote by the region.

**Figure 4:**
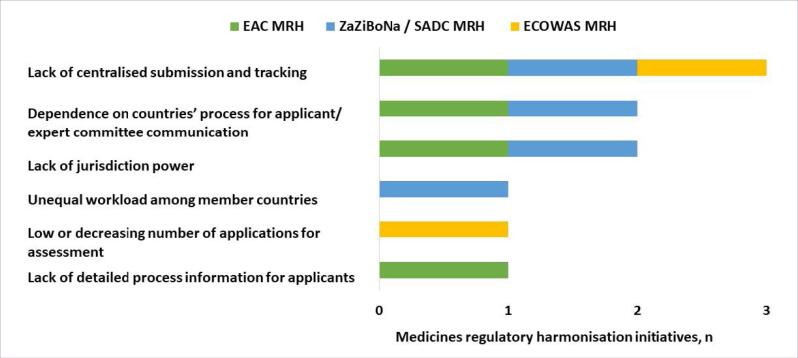
Weaknesses of the Medicines Regulatory Harmonisation Initiatives. Abbreviations: EAC, East African Community; ECOWAS, Economic Community of West African States; MRH, Medicines Regulatory Harmonisation; SADC, Southern African Development Community.

 There was consensus amongst all three regions that the lack of centralised submission and tracking was a weakness of the MRH initiatives. The dependence on the countries’ processes for communication with applicants and expert committees and the lack of jurisdiction power (the ability to mandate registration) were also identified as weaknesses by the EAC MRH and ZaZiBoNa/SADC MRH (Figure 4).

####  Challenges Faced at Country Level in Implementing the MRH Programme

 The three initiatives unanimously agreed that a challenge in implementing the MRH programme is inadequate human resources. Failure by manufacturers to follow the requirement to submit the exact same dossier to all countries of interest and to adhere to deadlines for responses to questions were additional challenges faced by the EAC MRH and the ZaZiBoNa/SADC MRH.

**Figure 5:**
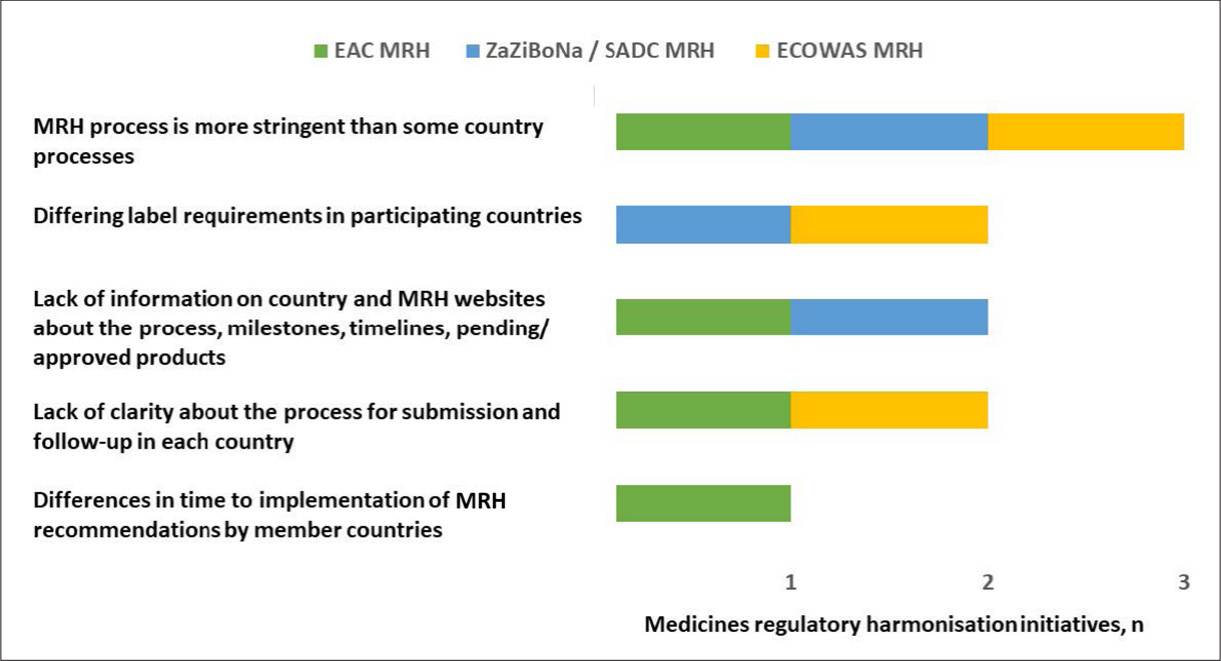
Challenges Faced by Applicants Submitting Applications to the Medicines Regulatory Harmonisation Initiatives. Abbreviations: EAC, East African Community; ECOWAS, Economic Community of West African States; MRH, Medicines Regulatory Harmonisation; SADC, Southern African Development Community.

 All three initiatives were of the view that a challenge faced by applicants is that the MRH process is more stringent than some country processes. Additional challenges faced by applicants identified by two of the three MRH initiatives were differing labelling requirements in participating countries, lack of information on country websites and the MRH website about the process, milestones, timelines and pending and approved products and a lack of clarity about the process for submission and follow-up in each country (Figure 5).

####  Accessibility and Affordability of Medicines

 An interesting finding from this study was the consensus amongst the three regions that although the MRH programmes had resulted in quicker access and increased availability of quality-assured medicines for patients, this was not necessarily at a reduced price. This could be because most of the regulatory authorities participating in these initiatives are not responsible for regulating the pricing of medicines; moreover, there are no health technology assessment agencies in these countries to perform this function as is the practice in other jurisdictions.^22^ As a result, the harmonisation of requirements and work sharing has not resulted in the availability of medicines at a lower price for patients; however, one way the regions plan to negotiate lower prices for medicines is through the implementation of pooled procurement.

###  Recommendations

 The following recommendations are based on the synthesis of the results by the authors, which were then endorsed by the regulatory authorities.

- *Aligning the operating models to improve efficiency:*The EAC MRH and ZaZiBoNa/SADC MRH should consider developing a framework to enable a centralised regional submission and review prior to submission to the individual countries of interest for registration as is the situation in the ECOWAS MRH. In addition, the two-year period given by the ECOWAS MRH for applicants to submit applications to the country after a regional review needs to be revised to align with the other two regions, EAC MRH and ZaZiBoNa/SADC MRH, in which registration in the individual countries is pursued immediately after the regional review.
- *Legal framework:* All three initiatives should consider using three routes/procedures for the approval of medical products in their regions; that is, a fully centralised procedure, a decentralised procedure and a national procedure. For all three regions, this would entail pursuing the development of a regional legally binding framework, if possible, to allow the establishment of a centralised procedure.
- *Communication with applicants: *The initiatives implementing any form of a decentralised procedure at submission; that is, EAC MRH and ZaZiBoNa/SADC MRH should communicate with existing and prospective applicants, the target timelines for the joint review process as well as to highlight that the timelines for approval in countries will differ and be dependent on the national process, as it is for other decentralised procedure such as that of the EMA or Australia-Canada-Singapore-Switzerland-United Kingdom Consortium.
- *Publishing an expression of interest:* The EAC MRH and ZaZiBoNa/SADC MRH should implement the practice of publishing an expression of interest as is the situation by the ECOWAS MRH.
- *Information management systems:*In the absence of legally binding frameworks, the regional economic communities (RECs) should invest in robust information management systems to address the weaknesses and challenges identified in this study such as the poor tracking of products and monitoring of timelines in the countries after a joint review is completed.
- *Reliance: *The RECs should continue to support and advocate the strengthening of the capacity of their member states using the WHO GBT assessments and other tools such as Optimising Efficiencies in Regulatory Agencies (OpERA) and Quality of Decision-Making Orientation Scheme (QoDoS) to facilitate inter-country and inter-REC reliance including unilateral and mutual recognition.

## Discussion

 The AMRH has made significant gains in the strengthening of national regulatory systems and the harmonisation of regulatory requirements since its formation in 2009. According to the regulatory authorities that participated in this study, the three registration harmonisation projects have all managed to meet the core objectives, which were to harmonise guidelines and registration requirements and to build the capacity of member states. The objectives of shorter timelines and simultaneous access to various markets have not been as straightforward to achieve for all the regions, as they are dependent on the time taken by the individual countries to issue a registration/marketing authorisation upon completion of the joint scientific review and in addition for EAC MRH and ECOWAS MRH the time taken by the applicant to submit an application for registration of a jointly reviewed product to the individual countries. The EMA, which has been in existence for over 25 years, provides a blueprint from which the regional harmonisation initiatives in Africa can learn.

 Registration or marketing authorisation of a medical product is a legal decision that can only be issued by a legally mandated entity, usually a national regulatory authority within a jurisdiction.^1^ As such, networks, organisations or entities without that legal mandate cannot issue a registration. Aware that this limitation existed in the RECs, EAC, ECOWAS and SADC, the regulators decided to establish their work-sharing initiatives as a decentralised model or a hybrid of the decentralised and centralised models, leaving the responsibility for issuing registrations to the national regulatory authorities in their respective countries. This decision has borne fruit, as we report the results of this study show that the initiatives have successfully developed regional guidelines and templates and conducted joint reviews of many products.^8,13,23^ The initiatives also resulted in building the capacity of member states; for example, in the EAC, Burundi, Rwanda, and Zanzibar were supported in the establishment of semi-autonomous national regulatory authorities that previously did not exist.^24^ In SADC, Angola, and Mozambique were also supported in the establishment of semi-autonomous national regulatory authorities. However, there has been some disappointment with the joint review initiatives for the pharmaceutical industry, as their expectation was to have a fully centralised process with a single approval enabling simultaneous access to various markets.^25^

 In hindsight, the simultaneous access should not have been promised or expected, as it can only be achieved in a fully centralised process with jurisdiction power, a situation currently not possible due to the founding and operating principles of the RECs. A better approach would have been to communicate the target timelines for the joint review process to applicants from the outset, while highlighting that the timelines for approval in countries would differ and be dependent on the national process as is carried out for the decentralised procedure of the EMA and other similar work-sharing initiatives such as the Australia-Canada-Singapore-Switzerland-United Kingdom Consortium.^26^ One initiative that can immediately be implemented to bring alignment in the operating models of the three initiatives and improve efficiency is for the EAC MRH and ZaZiBoNa/SADC MRH to develop a framework to enable a centralised regional submission and review prior to submission to the individual countries of interest for registration, as is carried out in the ECOWAS MRH. In addition, the two-year period given by the ECOWAS MRH for applicants to submit applications to the country after a regional review needs to be revised to align with the other two regions, EAC MRH and ZaZiBoNa/SADC MRH, in which registration in the individual countries is pursued immediately after the regional review. In addition, the lengthiness of this two-year period negates the benefit of shorter registration times that the MRH programme seeks to achieve.

 However, it is recommended that all three initiatives consider using three routes/procedures for the approval of medical products in their regions; that is, a fully centralised procedure, a decentralised procedure and a national procedure. For the three regions, this would entail pursuing the development of a regional legally binding framework, if possible, to allow the establishment of a fully centralised procedure as is carried out in the European Union. The use of the centralised procedure could be made mandatory for certain critical medical products to ensure equitable access in all member states, regardless of regulatory capacity or maturity. The use of regional experts in the assessment of complex products and central safety monitoring is another benefit of a centralised procedure.

 Investment in robust information management systems is critical to immediately address the additional weaknesses or challenges identified with the current operating models of the initiatives in this study such as the lack of detailed information for applicants on procedures and the lack of adequate tracking and monitoring of timelines for products in the participating countries once the joint review is completed. This investment will empower the region to publish this information for stakeholders, improving transparency and confidence in the process. This is supported by other studies conducted in these regions, which advocated greater transparency and the use of metrics to identify opportunities to improve efficiency.^27,28^

 From the results of this study, it is evident that the countries participating in the three RECs have successfully implemented reliance by leveraging the regulatory work of other NMRAs as well as regional reliance mechanisms. For example, several countries in the RECs have signed bilateral agreements to facilitate the sharing of information for abridged and verification reviews. There is potential for the countries to further implement reliance through unilateral and mutual recognition. Currently, in the East African region, Zanzibar unilaterally recognises the decisions of Tanzania; in the Southern African region, Eswatini, Mauritius and Namibia unilaterally recognise the decisions of South Africa. The regions should continue to support and advocate the strengthening of the capacity of their member states using the WHO GBT assessments (formal and informal). As capacity and trust is built, more countries will consider implementing unilateral and mutual recognition within a region as well as between the different RECs on the continent. In addition, measures should be implemented to increase efficiency in the regulatory review process such as the use of the OpERA tool to track, monitor and evaluate performance.^29^ Greater transparency through the publishing of public assessment reports as well as documenting the benefit-risk assessments conducted and the basis for reaching decisions using tools such as the QoDoS will facilitate a greater extent of reliance.^30^

###  Limitations and Future Work 

 The scope of this study was limited to the processes and operating models of the regional harmonisation initiatives. In future, it would be helpful to obtain quantitative data to support these views. For example, the specific metrics of the time taken to register the medicinal products in the individual countries after a regional recommendation and the status of commercialisation and pricing of the medicinal products in the individual countries as well as the factors influencing these metrics could be the subject of a future study.

## Conclusion

 This study has highlighted the successes of the medicine registration harmonisation initiatives in Africa as well some opportunities for improvement and alignment. The results of this comparison allow for the three regional harmonisation initiatives to learn from each other, and the implementation of the recommendations made in this study will bring greater alignment and efficiency in their operating models thereby strengthening the foundation of the soon to be operationalised AMA.

## Ethical issues

 The study was approved by the Health, Science, Engineering and Technology ECDA, University of Hertfordshire, United Kingdom [Reference Protocol number: LMS/PGR/UH/04988]. Data were managed in compliance with the General Data Protection Regulation and any regulations regarding management of personal data required by participants’ respective country of residence. All the national medicine regulatory authorities in East Africa approached to take part in the study were satisfied with ethics approval obtained from the United Kingdom and did not require us to apply for any IRBs in East Africa.

## Competing interests

 Authors declare that they have no competing interests.

## Data availability statement

 We include a copy of the questionnaire in the Supplementary materials and the raw data will be available on request.

## Funding

 This study was supported by an unrestricted grant from the Bill and Melinda Gates Foundation.

## Supplementary files

Supplementary file 1 contains Box 1.

## References

[1] Rägo L, Santoso B. Drug regulation: history, present and future. In: van Boxtel CJ, Santoso B, Edwards IR, eds. Drug Benefits and Risks: International Textbook of Clinical Pharmacology. 2nd ed. Uppsala: IOS Press, Uppsala Monitoring Centre; 2008.

[2] European Medicines Agency. EMA Starts First Rolling Review of a COVID-19 Vaccine in the EU. https://www.ema.europa.eu/en/news/ema-starts-first-rolling-review-covid-19-vaccine-eu. Accessed January 30, 2024.

[3] World Health Organization (WHO). TRS 1033 - Annex 10: Good Reliance Practices in the Regulation of Medical Products: High Level Principles and Considerations. WHO Expert Committee on Specifications for Pharmaceutical Preparations: Fifty-Fifth Report. WHO. 2021:237-267.

[4] World Health Organization (WHO). Essential medicines and health products. WHO Global Benchmarking Tool (GBT) for Evaluation of National Regulatory Systems. 2021. https://www.who.int/tools/global-benchmarking-tools/VI. Accessed January 30, 2024.

[5] McAuslane N, Bujar M, Sithole T, Ngum N, Owusu-Asante M, Walker S (2023). Evaluation of risk-based approaches to the registration of medicines: current status among African regulatory authorities. Pharmaceut Med.

[6] Ndomondo-Sigonda M, Miot J, Naidoo S, Ambali A, Dodoo A, Mkandawire H (2018). The African Medicines Regulatory Harmonization Initiative: progress to date. Med Res Arch.

[7] Sillo H, Ambali A, Azatyan S (2020). Coming together to improve access to medicines: the genesis of the East African Community’s Medicines Regulatory Harmonization initiative. PLoS Med.

[8] Ngum N, Mashingia J, Ndomondo-Sigonda M, Walker S, Salek S (2022). Evaluation of the effectiveness and efficiency of the East African Community joint assessment procedure by member countries: the way forward. Front Pharmacol.

[9] World Health Organization (WHO). Tanzania Food and Drug Authority Becomes the First to Reach Level 3 of the WHO Benchmarking Programme. WHO; 2018. https://www.afro.who.int/news/tanzania-food-and-drug-authority-becomes-first-reach-level-3-who-benchmarking-programme. Accessed January 30, 2024.

[10] Sithole T, Mahlangu G, Salek S, Walker S (2020). Evaluating the success of ZaZiBoNa, the Southern African development community collaborative medicines registration initiative. Ther InnovRegul Sci.

[11] World Health Organization (WHO). South Africa’s Vaccine Regulator Reaches New WHO Level to Ensure Safety, Quality and Effectiveness. WHO; 2022. https://www.who.int/news/item/05-10-2022-south-africa-s-vaccine-regulator-reaches-new-who-level-to-ensure-safety-quality-effectiveness#:~:text=The%20designation%20Maturity%20Level%203,performance%20and%20with%20continuous%20improvement. Accessed January 30, 2024.

[12] Daniel E. Harmonising Medicines’ Regulation in West Africa. The Guardian; 2019. Available at: https://guardian.ng/features/harmonising-medicines-regulation-in-west-africa/. Accessed January 30, 2024.

[13] Owusu-Asante M, Darko DM, Walker S, Salek S (2022). Assessment of the effectiveness and efficiency of the West Africa Medicines Regulatory Harmonization initiative by the member countries. Front Pharmacol.

[14] Economic Community of West African States (ECOWAS). Regional Joint Assessment Procedure for Medicine Registration and Marketing Authorization of Medicinal Products. West African Health Organization (WAHO); 2019. https://www.wahooas.org/web-ooas/sites/default/files/publications/1993/wa-mrh-regional-joint-medicines-assessment-procedure.pdf. Accessed January 30, 2024.

[15] Barton I, Avanceña AL, Gounden N, Anupindi R (2019). Unintended consequences and hidden obstacles in medicine access in sub-Saharan Africa. Front Public Health.

[16] Adler RH (2022). Trustworthiness in qualitative research. J Hum Lact.

[17] Gunawan J (2015). Ensuring trustworthiness in qualitative research. Belitung Nurs J.

[18] Ul Haq Kakar Z, Rasheed R, Rashid A, Akhter S (2023). Criteria for assessing and ensuring the trustworthiness in qualitative research. Int J Bus Reflect.

[19] Howitt D, Cramer D. Introduction to Research Methods in Psychology. 2nd ed. New York: Pearson Education Ltd; 2008.

[20] Charmaz K. Constructing Grounded Theory: A Practical Guide Through Qualitative Analysis. Thousand Oaks: SAGE Publications; 2006.

[21] Spencer L, Ritchie J, O’Connor W, Morrell G, Ormston R. Analysis in practice. In: Ritchie J, Lewis J, eds. Qualitative Research Practice: A Guide for Social Science Students and Researchers. London: SAGE Publications; 2014.

[22] Sithole TD. An Evaluation of the Regulatory Review System in the Southern African Development Community Work Sharing Initiative (ZaZiBoNa): Enhancing the Review Process and Patients’ Access to Medicines [thesis]. Hatfield: University of Hertfordshire; 2022.

[23] Sithole T, Mahlangu G, Walker S, Salek S (2022). Regulatory authority evaluation of the effectiveness and efficiency of the ZaZiBoNa collaborative medicines registration initiative: the way forward. Front Med (Lausanne).

[24] East African Community (EAC). East African Community Stakeholder’s Consultative Meeting on EAC Joint Regulatory Procedure and Sustainability Plan. EAC; 2022. https://www.eac.int/news-and-media/calendar-of-events/event/891-stakeholder%E2%80%99s-consultative-meeting-on-eac-joint-regulatory-procedure-and-sustainability-plan. Accessed January 30, 2024.

[25] Dansie LS, Odoch WD, Årdal C (2019). Industrial perceptions of medicines regulatory harmonization in the East African Community. PLoS One.

[26] Australian Government Department of Health and Aged Care, Therapeutic Goods Administration. Australia-Canada-Singapore-Switzerland-United Kingdom (ACCESS) Consortium. https://www.tga.gov.au/international-activities/australia-canada-singapore-switzerland-united-kingdom-access-consortium. Accessed January 30, 2024.

[27] Giaquinto AR, Grignolo A, Liberti L (2020). Improving access to quality medicines in East Africa: an independent perspective on the East African Community Medicines Regulatory Harmonization initiative. PLoS Med.

[28] Sithole T, Salek S, Mahlangu G, Walker S (2022). Comparison of the registration process of the medicines control authority of Zimbabwe with Australia, Canada, Singapore, and Switzerland: benchmarking best practices. Expert Rev Clin Pharmacol.

[29] Sithole T, Mahlangu G, Salek S, Walker S (2021). Evaluation of the regulatory review process in Zimbabwe: challenges and opportunities. Ther InnovRegul Sci.

[30] Bujar M, McAuslane N, Walker S, Salek S (2019). The reliability and relevance of a quality of decision-making instrument, Quality of Decision-Making Orientation Scheme (QoDoS), for use during the lifecycle of medicines. Front Pharmacol.

