# Supplementary material for: Comparison of Three Regional Medicines Regulatory Harmonisation Initiatives in Africa: Opportunities for Improvement and Alignment
Source: Int J Health Policy Manag. 2024 May 5;13:8070. doi: 10.34172/ijhpm.2024.8070 (PMC11270597; doi:10.34172/ijhpm.2024.8070)
Supplement: Supplementary file 1 — contains Box 1. [file ijhpm-13-8070-s001.pdf]

Supplementary Box 1

EVALUATION OF THE PEER QUESTIONNAIRE

Content Validity

(Including cognitive debriefing)

As part of our goal to make sure that the questionnaires we have developed as an assessment tool for our research project is meeting the objectives of our studies, we would be most grateful for your feedback to help us to improve this ZAZIBONA PEER questionnaire. So would you be kind enough, once you have completed the PEER questionnaire to answer the following by selecting the appropriate tick box:

1. Did you find the questions clear and straightforward to respond to? Yes ☐ No ☐
2. Did you find the response options relevant to the heading of each section (A to E)?...Yes ☐ No ☐
3. Did you find the questions relevant to the aims and objectives of the study?...Yes ☐ No ☐
4. Did you find the questions relevant to your authority and ZAZIBONA work sharing initiative?...Yes ☐ No ☐
5. Did you find any relevant questions missing?....................................Yes ☐ No ☐

   If yes, please state which questions were missing in the space after this list of questions.
6. Did you find any questions that should be excluded?.........................Yes ☐ No ☐

   If yes, please state the questions that should be excluded in the space after this list of questions.
7. Did you find the questionnaire useful to reflect on both your agency experience as well that of ZAZIBONA?...Yes ☐ No ☐

Name:____

Agency:__________
